# Global Cardiothoracic Surgery in an Academic Career: Lessons from Brazil

**DOI:** 10.21470/1678-9741-2023-0962

**Published:** 2023-07-18

**Authors:** Vinicius Nina, Emily Farkas, DuyKhanh Ceppa, Aubyn Marath

**Affiliations:** 1 Department of Medicine I, Curso de Medicina, Universidade Federal do Maranhão, São Luís, Maranhão, Brazil; 2 Division of Cardiothoracic Surgery, Indiana University School of Medicine, Indiana, United States of America; 3 CardioStart International, Florida, United States of America

According to the legendary physician, educator, and medical statesman Dr. Michael E. DeBakey, an academic surgeon is a physician-scientist who typically devotes years of careful observation, analysis, and iterative investigation to identify and solve challenging or unexplored clinical problems, and then he/she employs available resources in his/her medical community to support these endeavors. Dr. Fred A. Crawford Jr., Distinguished Professor of Surgery at the Medical University of South Carolina, has cut this definition short stating that academic surgeons are “triple threat” surgeons who operate, teach, and also do research^[1]^.

In this regard, the Department of Surgery at Houston’s Baylor College of Medicine have also identified seven common attributes of the academic surgeon^[1]^:

1. Identifies complex clinical problems ignored or thought unsolvable by others
2. Becomes an expert
3. Innovates to advance treatment
4. Observes outcomes to further improve and innovate
5. Disseminates knowledge and expertise
6. Asks important questions to further improve care
7. Trains the next generation of surgeons and scientists

In our view, an eighth attribute should be added to this list - “Pay it forward by joining/leading collaborative and cooperative initiatives”, because the world is desperate for more pairs of skilled hands^[1,2]^.

This need is well documented in the Key Messages from The Lancet Commission on Global Surgery^[3]^:

- Five billion people lack access to safe, affordable surgical and anesthesia care
- 143 million additional procedures are needed yearly
- 33 million people face catastrophic expense after surgical care yearly
- Investment in surgical and anesthesia care saves lives, is affordable, and promotes economic growth
- Surgery is an indivisible, indispensable part of health care

Access to cardiac surgical care around the world is not different from other surgical fields. According to Vervoort et al., 4.5 billion people lack access to cardiac surgery when needed. Availability of an adult and pediatric cardiac surgical workforce is scarce in low-and middle-income countries, and disparities are widespread^[4]^.

Academic Surgeons *cannot* ignore those facts! Because as the most influential physicians they must make the difference by identifying new opportunities for improving patient care wherever it is needed.

“It isn’t enough to think outside the box. Thinking is passive. Get used to acting outside the box.”

Tim Ferriss, American entrepreneur.

An academic surgeon can act outside his/her own arena by cooperating with humanitarian initiatives in taking leadership positions or simply by participating as a volunteer in a surgical mission. There are different ways to contribute onsite: discussion on rounds, wet labs, during surgery (“teaching cases”), lectures and informal chatting while socializing, or even during relaxing moments with the local team^[5]^.

However, there are some desirable and/or expected qualities of an academic cardiothoracic surgeon working in the humanitarian sector. These qualities include:

- Charisma
- Honesty
- Integrity
- Focus on mentorship
- Timely decision-making
- Effective communication
- Collegiality & the ability to be a team player
- Respect for local norms, priorities, and customs
- Acknowledgment of responsibility toward patients, team, & multidisciplinary local providers^[5]^

Can an academic cardiothoracic surgeon possess all of that magic? The answer is yes, because of the power of our commitment to address complex problems and to embrace challenges for the sake of our patients, wherever they are.

“There are certain individuals who choose certain careers because of a powerful calling to do so. They would do what they like to do even if they were not paid for it, just for the sake of doing the job. These unique individuals simply love to work. They find their jobs interesting, challenging, and fulfilling. Most surgeons are like that.”^[6]^

Tirone E. David, MD

David TE. Innovation in Surgery. J Thorac Cardiovasc Surg. 2000;119:S38-41.

Such commitment has allowed us to learn two important lessons:

1. One of the major requirements for combining an academic career and humanitarian work is to make the time available. This will require a fairly delicate balancing act between work, family, outside interests, and organizational activities.
2. The combination of those, however, provides immense personal gratification because the experiences and memories from both continue to shape your practice and your soul, and the stories you tell may inspire others to enter academia and participate in medical missions as well.

Therefore, the rewards of being an academic cardiothoracic surgeon allow one to mold the future of the specialty *wherever* it is needed.
